# Supplementary material for: Integrating single-cell RNA-seq and imaging with SCOPE-seq2
Source: Sci Rep. 2020 Nov 10;10:19482. doi: 10.1038/s41598-020-76599-w (PMC7655825; doi:10.1038/s41598-020-76599-w)
Supplement: Supplementary file 1 — Supplementary Information 1. [file 41598_2020_76599_MOESM1_ESM.pdf]

Supplementary Materials

Integrating single-cell RNA-seq and imaging with SCOPE-seq2

Zhouzerui Liu[1*], Jinzhou Yuan[1*], Anna Lasorella[2,3,4], Antonio Iavarone[2,4,5], Jeffrey N. Bruce[6], Peter Canoll[4] and Peter A. Sims[1,7,8,#]

1. Department of Systems Biology, Columbia University Irving Medical Center, New York, NY 10032, USA.
2. Institute for Cancer Genetics, Herbert Irving Comprehensive Cancer Center, Columbia University Irving Medical Center, New York, NY 10032, USA
3. Department of Pediatrics, Columbia University Irving Medical Center, New York, NY 10032 USA
4. Department of Pathology & Cell Biology, Columbia University Irving Medical Center, New York, NY 10032 USA
5. Department of Neurology, Columbia University Irving Medical Center, New York, NY 10032 USA
6. Department of Neurological Surgery, Columbia University Irving Medical Center, New York, NY 10032, USA
7. Department of Biochemistry & Molecular Biophysics, Columbia University Irving Medical Center, New York, NY 10032, USA
8. Sulzberger Columbia Genome Center, Columbia University Irving Medical Center, New York, NY 10032, USA

# Correspondence to: pas2182@cumc.columbia.edu

* Equal contribution.

Table Legends

Supplementary Table 1. Cell barcode sequences.

Supplementary Table 2. Fluorescent probe sequences for cell barcode optical decoding.

Supplementary Table 3. Cell barcode temporal binary codes.

Supplementary Table 4. Statistical analysis comparing the imaging features of cellular subpopulations identified by scRNA-seq.

Supplementary Table 5. Differential expression of two imaging clusters.

Supplementary Table 6. Primer sequences.

Source Data Legends

Source Data for Figure 4. Gene score matrix from the scHPF model of all GBM cells.

Source Data for Figure 4C. Z-scored cell imaging features.

Source Data for Figure 5. Gene score matrix from the scHPF model of malignantly transformed GBM cells.

Supplementary Figures

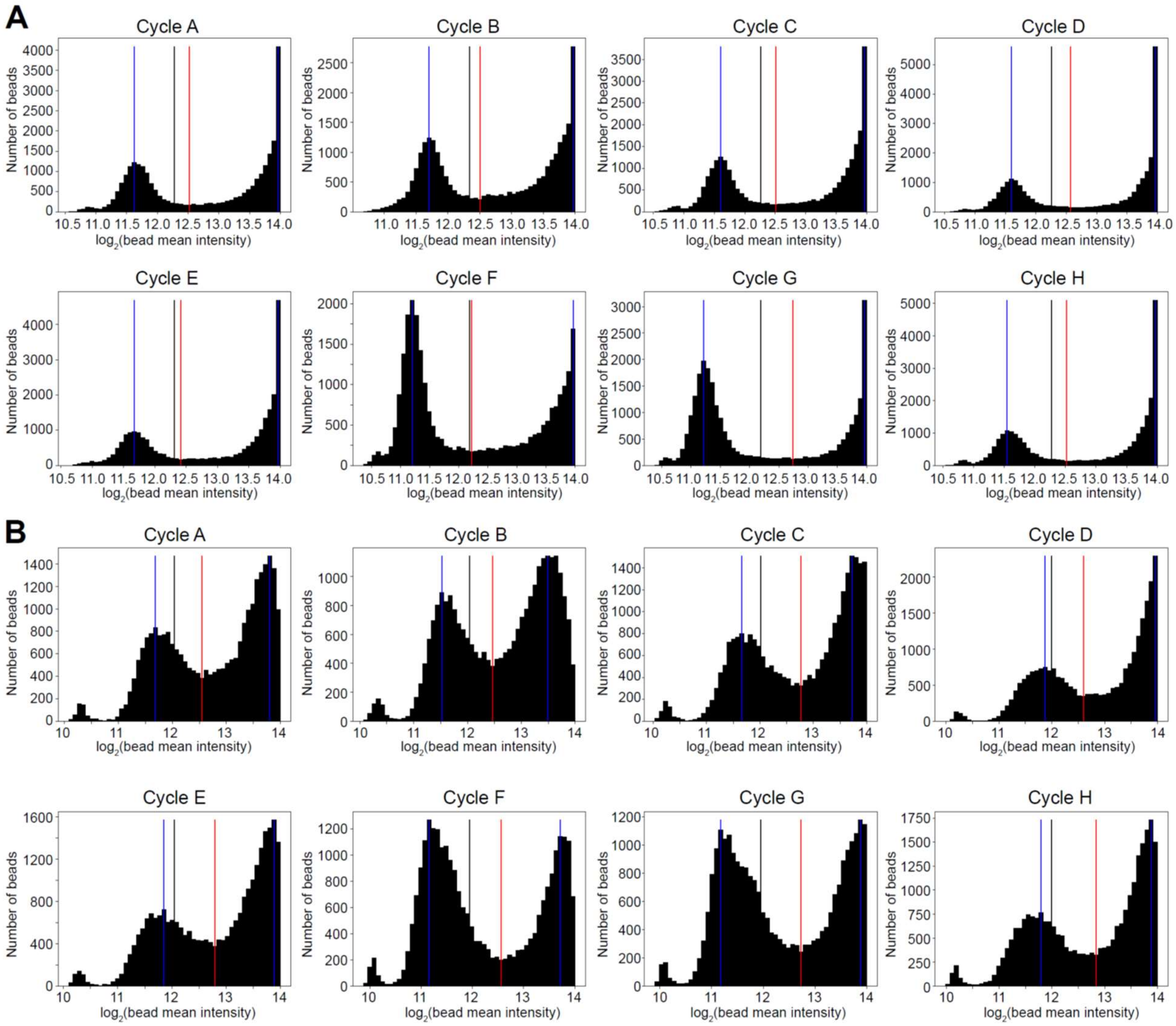

Supplementary Figure 1 - Cell barcode optical decoding. A), B) Histograms show fluorescence intensity distributions of probe hybridizations for cell barcode S, which are labeled with Cy5 (A) and for cell barcode Q, which are labeled with Cy5 (B) in data set PJ070. Black vertical line shows the median bin; blue vertical line shows the highest bins on each side of the black vertical line; red vertical line shows the lowest bin, the threshold to separate 'zero' and 'one' populations, between the two blue vertical lines.

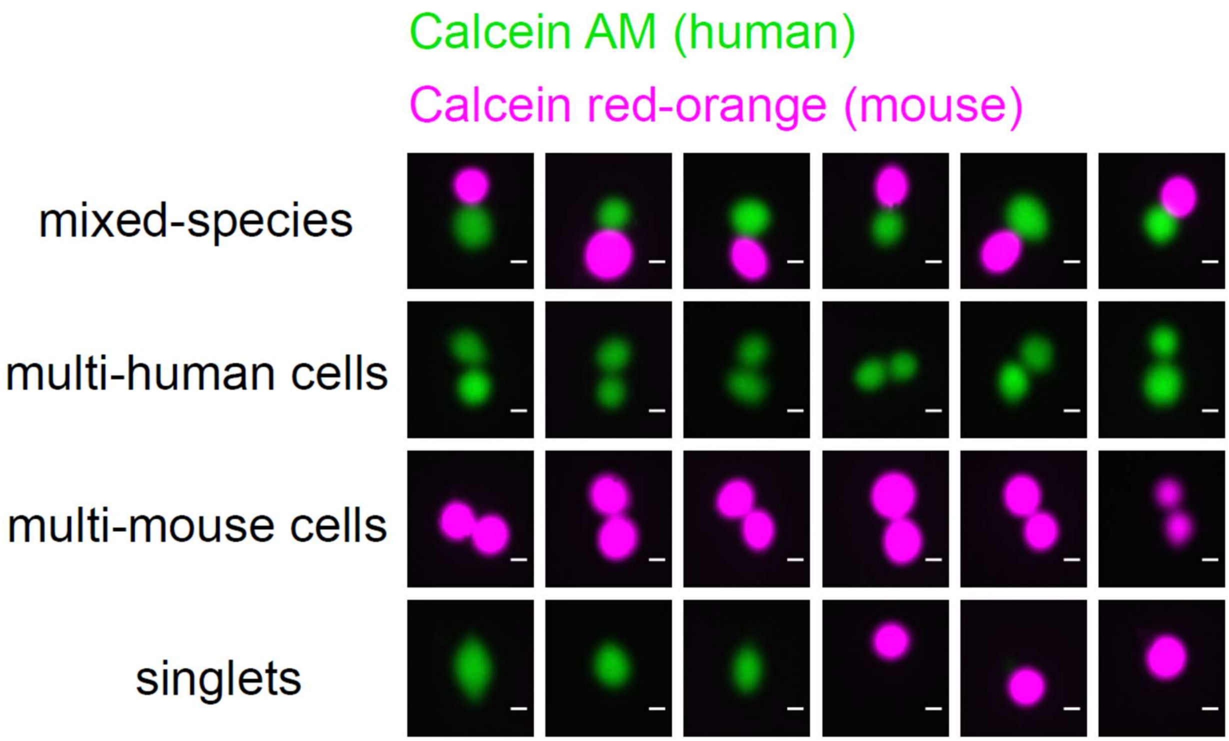

Supplementary Figure 2 - Multiplet detection. Fluorescence images show example wells with mixed-species cells, multi-human cells, multi-mouse cells and singlets. Scale bar: 10 µm.

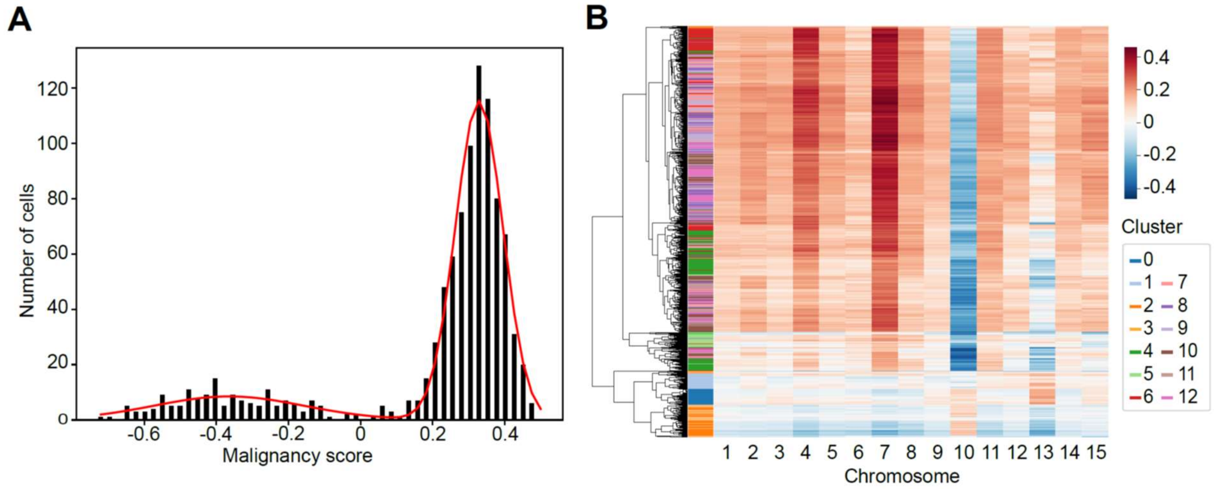

Supplementary Figure 3 – Malignantly transformed GBM cell identification. A) Histogram shows the distribution of malignancy scores across all cells. B) Heatmap shows the relative chromosomal average expression (chromosomal average expression with subtracted non-malignant cell chromosome average expression subtracted). Colors indicate the Phenograph cell clusters.
